# Lung Cancer Mortality Trends in China from 1988 to 2013: New Challenges and Opportunities for the Government

**DOI:** 10.3390/ijerph13111052

**Published:** 2016-10-28

**Authors:** Lijun Wang, Chuanhua Yu, Yu Liu, Jun Wang, Chunhui Li, Quan Wang, Peigang Wang, Shaotang Wu, Zhi-Jiang Zhang

**Affiliations:** 1 Department of Epidemiology and Biostatistics, School of Public Health, Wuhan University, No. 185 Donghu Road, Wuhan 430071, China; wanglj1003@163.com (L.W.); yuchua@163.com (C.Y.); 2 Global Health Institute, Wuhan University, No. 185 Donghu Road, Wuhan 430071, China; wangquan73@whu.edu.cn (Q.W.); wpg926@163.com (P.W.); tangdream@whu.edu.cn (S.W.); 3 Department of Statistics and Management, School of Management, Wuhan Institute of Technology, 206 Optical Valley Avenue, Wuhan 430205, China; lyu429@163.com; 4 Institute of Health Finance and Economics, Central University of Finance and Economics, 39 Xueyuan South Road, Beijing 100081, China; sfdrcufe@126.com; 5 Institute of National Health and Development, Shanghai University of Finance and Economics, 777 Guoding Road, Shanghai 200433, China; 6 School of Public Health, Dalian Medical University, No. 9 Lvshun South Road, Dalian 116044, China; chli0201@hotmail.com; 7 Department of Social Medicine and Health Management, School of Public Health, Wuhan University, No. 185 Donghu Road, Wuhan 430071, China

**Keywords:** age-period-cohort models, lung cancer mortality, air pollution, medical security, tobacco control

## Abstract

*Background*: As lung cancer has shown a continuously increasing trend in many countries, it is essential to stay abreast of lung cancer mortality information and take informed actions with a theoretical basis derived from appropriate and practical statistical methods. *Methods*: Age-specific rates were collected by gender and region (urban/rural) and analysed with descriptive methods and age-period-cohort models to estimate the trends in lung cancer mortality in China from 1988 to 2013. *Results*: Descriptive analysis revealed that the age-specific mortality rates of lung cancer in rural residents increased markedly over the last three decades, and there was no obvious increase in urban residents. APC analysis showed that the lung cancer mortality rates significantly increased with age (20–84), rose slightly with the time period, and decreased with the cohort, except for the rural cohorts born during the early years (1909–1928). The trends in the patterns of the period and cohort effects showed marked disparities between the urban and rural residents. *Conclusions*: Lung cancer mortality remains serious and is likely to continue to rise in China. Some known measures are suggested to be decisive factors in mitigating lung cancer, such as environmental conservation, medical security, and tobacco control, which should be implemented more vigorously over the long term in China, especially in rural areas.

## 1. Introduction

In recent years, with the changing of the human disease spectrum, malignant tumors have been one of the most severe diseases to threaten people’s health and one of the most acute problems that urgently needs to be settled all over the world. The incidence of cancer is increasing because of the growth and ageing of the population, spread of established risk factors such as air pollution, smoking, physical inactivity [1], as well as lack of proven effective chemoprevention agents though promising [2,3]. Historically, there have been more cancer cases in developed countries than in less developed countries, but this trend is not permanent, and the burden of cancer will continue to shift to less developed countries due to the growth and ageing of the population and a cumulative prevalence of known risk factors [1]. The latest estimation of Global Burden of Cancer 2013 published that tracheal, bronchus, and lung (TBL) cancer was the leading cause of cancer death for both males and females, with 1.6 million deaths (age-standardized death rates, 27.0 per 100,000) [4]. In China alone, there were 546,259 TBL cancer deaths, about a third of the 1,639,646 deaths on a global scale in 2013 [4]. 

To better understand the national trends in lung cancer in China, many relevant studies have been conducted, and most of the studies described an increasing trend. Chen et al. [5] found a 1.63% increase of lung cancer incidence per year from 1988 to 2005, and the trend slowed down slightly after adjusting for age. This trend was also found by Fang et al. [6] with a 7.7% increase per year and a continued increase in the ensuing five years. The age-period-cohort (APC) analysis has been used in China for a few years, and some research has been performed on lung cancer. The results of Chen et al. [7] indicated no obvious change of period and cohort effects for a relatively short time from 1998 to 2007, and Du et al. [8] identified an increased trend in both males and females in Sihui City, China from 1987 to 2011. However, there is little understanding of the independent effects of life-course, time periods, and birth cohorts on lung cancer mortality with a large-scale and long-duration data set in China, and this will lead to bias regarding the observed trends.

The pioneers of the APC model are Mason et al. [9] who laid out the multiple classification framework, which gave a general functional form to estimate the independent effects of age, period and cohort and played an important role in many fields, such as sociology, demography and epidemiology. Age is the most important source of variations in vital rates, based on the biological theories that mortality risk increases with the process of ageing. Period effects represent influential factors that simultaneously affect all age groups. For example, complex sets of historical events and environmental factors—such as world wars, economic crises, famine, infectious disease pandemics and medical technology breakthroughs—can lead to changes in mortality rates for individuals of all ages. Cohort effects represent variations across groups of individuals born in the same year or years. These variations may arise when each succeeding cohort carries the imprints of physical and social exposures from gestation to old age; these effects, such as the imprints of lifelong accumulation of exposure to risk factors, influence morbidity and mortality risks in specific ways [10]. APC analysis offers an important way to decompose trends in lung cancer mortality rates and an opportunity to form hypotheses regarding effective measures to address lung cancer in China.

So far, there is no national APC analysis for the secular data of lung cancer, much less a reasonable understanding of the data. Therefore, we are eager to answer some of these questions. First, do age, period, and birth cohort matter in the epidemic after being taken independently? Second, do disparities of age, period, and cohort effects exist between urban/rural residents and male/female residents considering the large gap of influential factors in lung cancer for these residents? Third, what could the government do to effectively control lung cancer mortality rates? As lung cancer is one of the most controllable cancers, our aim is to update the three-dimensional trends of age, period, and cohort, and provide theoretical support for effective action plans.

## 2. Materials and Methods

The analysis was carried out based on data from the WHO Cancer Mortality Database (International Agency for Research on Cancer, IARC, Lyon, France) and China Public Health Statistical Yearbooks in which the mortality data of Chinese residents were aggregated and stratified by gender, 5-year age group, and region (urban/rural). The reasons for using this data set are several: first, both databases were created by the Centre of Health Information and Statistics (CHIS) of China so that they were considered to be merged to reach a longer time span. The CHIS reporting system is an annual disease surveillance system which was set up in 1973 and based upon a 10% sample of the population (between 100 and 120 million persons) [11,12]. Sex and region (urban/rural) specific age-standardized mortality rates from the CHIS dataset were close to those from the (1990–1992) national survey, and the results of CHIS are published by WHO [13]. Second, mortality rates reflect lung cancer burden better than incidence rates with less missing cases, and will not rise with detection rates. Third, data from six time points—1988, 1993, 1998, 2003, 2008 and 2013 (the most recently available data)—were collected at 5-year intervals consistent with the age groups; this approach was used to prevent overlapping of the birth cohorts, which may obscure the net effects of the cohorts. Fourth, the group above 85 years old involved those aged 90 and older and did not meet the requirements of the APC models, and younger groups under 20 presented very low probabilities. Therefore, only thirteen age groups (20–24, 25–29, …, 80–84) were included in this analysis.

The analysis mainly contained a description of age-specific mortality rates and APC analysis. The APC model is essentially categorized into multiple regression models and can be expressed as:

(1) $$Y=\mu +\alpha X_{1}+\beta X_{2}+\gamma X_{3}+\varepsilon$$

where $X_{1}$, $X_{2}$, and $X_{3}$ denote age, period and cohort, respectively, and α,β,γ denote their parameter estimates, respectively.

When it refers to the estimation of cancer mortality rates, the model can be written as a log-linear Poisson model:

(2) $$ln[E(M_{ij})]=ln\left(D_{ij}/P_{ij}\right)=\mu +\alpha_{i}+\beta_{j}+\gamma_{k}+\varepsilon_{ij}$$

where $E(M_{ij})$ denotes the expectation of death rate for the ith age group for i=1,…,a age group at the jth time period for j=1,…,p time period born in the kth cohort for k=1,…, a+p−1 birth cohort of observed data; $M_{ij}$, $D_{ij}$ and $P_{ij}$ denote the observed death rate, death number and size of population of that group, respectively; μ denotes the intercept or adjusted mean death rate; $\alpha_{i}$ denotes the ith row age effect or the coefficient for the ith age group; $\beta_{j}$ denotes the jth column period effect or the coefficient for the jth time period; $\gamma_{k}$ denotes the kth diagonal cohort effect or the coefficient for the kth cohort; $\varepsilon_{ij}$ denotes the random error with expectation $E\left(\varepsilon_{ij}\right)=0$. Regression models (1) and (2) can be treated as fixed-effects generalized linear models after a reparameterization to center the parameters:

(3) $$\underset{i}{\sum }\alpha_{i}=\underset{j}{\sum }\beta_{j}=\underset{k}{\sum }\gamma_{k}=0$$

This treatment is equivalent to selecting an arbitrary point from each of the age, period, and cohort groups as a reference point. For example, for urban males, we set the mean of the age effects as the reference, and thus, the coefficients of the age effects indicated the relative effects of each age group compared with this mean. Therefore, the coefficients of the reference groups—$\alpha_{mean},\;\beta_{mean},\;\gamma_{mean}$—equalled zero.

Goodness-of-fit statistics were calculated and used to select the best-fitting models with several model selection criteria: models with the same *df* (degree of freedom), smaller deviance values denoting a higher degree of fit, and smaller values for Akaike’s Information Criterion (AIC) and Bayesian Information Criterion (BIC) with parameter penalty terms denoting a better fit.

In Formulas (1) and (2), the birth cohort can be expressed by age and period; that is, cohort = period − age, which leads to an infinite number of possible solutions to this model. Since Mason’s multiple classification framework, this non-identification problem has become a research focus of APC analysis. The conventional approaches to solving APC models include two-factor models and constrained generalized linear models (CGLIMs). Two-factor models are built with two of the factors in pairs—age-period (AP) models, age-cohort (AC) models, and period-cohort (PC) models—to eliminate collinearity. CGLIMs obtain unique solutions by imposing additional constraints on the parameters in the generalized linear models. The constraints are better suited to relying on external or side information because different constraints lead to different estimates of change across ages, periods, and cohorts. In addition to the reparameterization of Equation (3), we set the effect coefficients of the first two cohorts as equal ($\gamma_{1}=\gamma_{2}$), considering that our primary data analysis showed minor variations in the first several coefficients of cohorts.

A newer method is the intrinsic estimator (IE), described and evaluated by Fu and Yang et al. [14,15], which is based on estimable functions and the singular value decomposition of matrices. This group related the IE coefficients to those of conventional constrained APC models and showed that both yielded similar estimates of age, period, and cohort effects; the estimates obtained by the IE were more direct and did not require prior information to select appropriate model constraints [16]. Nonetheless, considering the concerns raised by O’Brien [17] and Pelzer [18] that the IE may be viewed as a type of conventional constrained solution and may yield non-uniqueness, we used the IE as well as other two approaches—CGLIMs and two-factor models—and selected the best-fitting models to process the data.

## 3. Results

### 3.1. Descriptive Analysis of Age-Specific Mortality Rates of Lung Cancer

**Figure 1:**
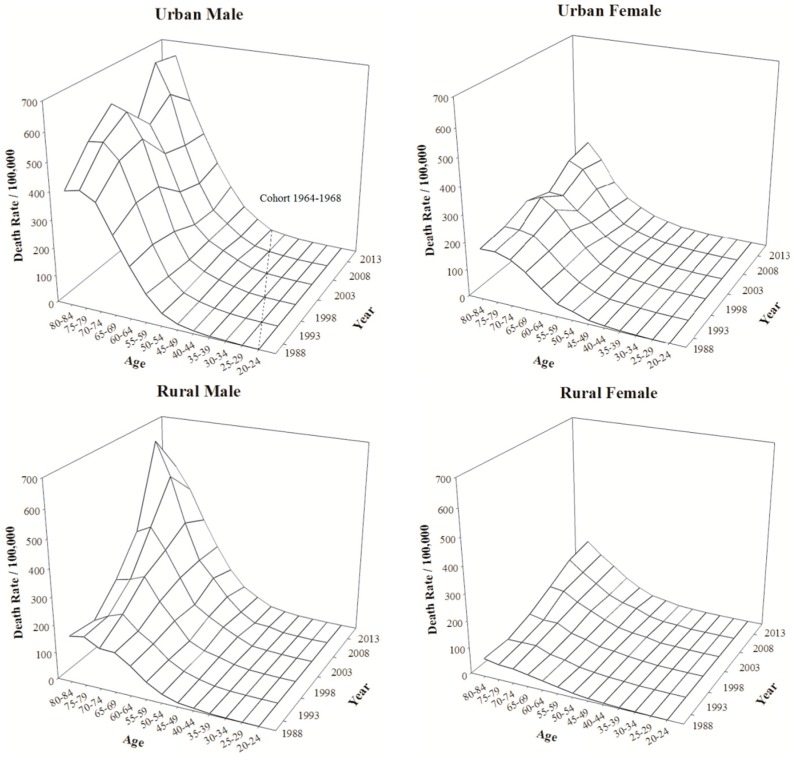
Lung cancer age-specific mortality rates (per 100,000 persons) for urban males, urban females, rural males, and rural females from 1988 to 2013 in China.

Figure 1 shows the age-specific annual lung cancer mortality rates (per 100,000 people) for Chinese residents aged 20–84 from 1988 to 2013, which provides us a direct impression of the past situation. Large gaps indicated that the rates were notably higher for males and urban residents than for females and rural residents, respectively, as many researchers have reported. The patterns of variation with age in the four populations were similar: the rates remained extremely low under 50 years of age and escalated sharply from age 50 to 70, after which the increase slowed down and the rates appeared to exhibit a slight decline in the elderly. The mortality rates of the 80–84 age group were lower than those of the 75–79 group among urban males in 1988, 1993, and 2003, urban females in 1998, and rural males in 1988, 1993, 1998, and 2003. The mortality rates of rural residents increased markedly with time, but the trends were not as apparent for urban residents. The diagonal elements of the mesh figure correspond to cohorts *k* = *i* + *j* − 1. For example, the dashed line shows the age and time variations in the 1964–1968 cohort. However, this figure contains all three effects simultaneously and is thus unable to show which temporal dimensions contribute to these trends.

### 3.2. Model Comparison

To estimate the three independent effects and changes across age, time periods, and birth cohorts, we fitted two-factor models and three-factor models and chose the best-fitting models to conduct the APC analysis. The two-factor models were fitted with Poisson log-linear model. The three-factor models were APC models solved with a conventional constrained Poisson log-linear model estimator (called APC-C) and the intrinsic estimator (called APC-IE). Goodness-of-fit was evaluated synthetically with deviance, AIC, and BIC, as shown in Table 1. The results indicated that the APC-IE models provided the best fit compared with the two-factor models and APC-C models; thus, the APC-IE models were selected for use in analyses of the collected data.

### 3.3. Age-Period-Cohort Analysis

**Figure 2:**
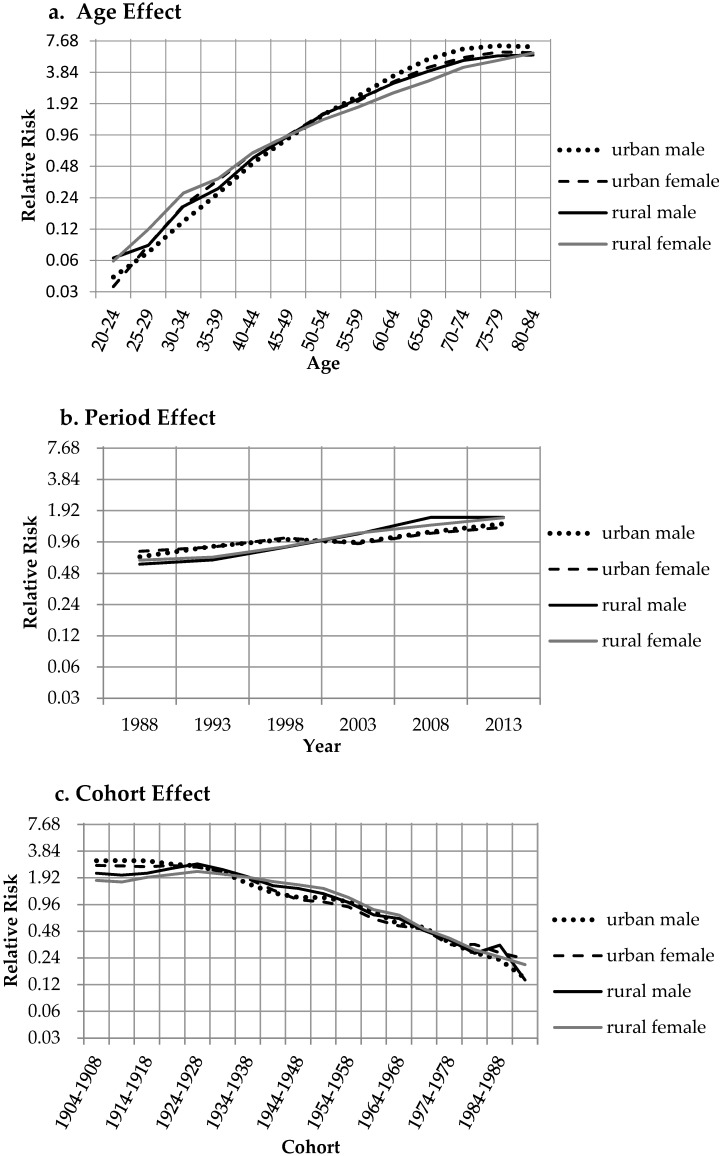
Lung cancer mortality relative risks due to (**a**) age; (**b**) period; and (**c**) cohort effects.

The results of the APC-IE models provided coefficients for successive categories within the age, time period and cohort classifications. The net age, period and cohort trends in mortality risk are shown in Table 2 and Figure 2. The regression coefficients (α,β, γ) in Formula (2) were calculated on a log scale—exp(α), exp(β), exp(γ)—and were interpreted as relative risks (RRs), reflecting the relative effects of a particular age, period, or cohort group compared with the average levels for the corresponding dimension. For example, for the period effect in urban males, the RR of lung cancer mortality in 2013 was $\exp\left(\beta_{2013}-\beta_{mean}\right)=\exp\left(\beta_{2013}\right)\approx 1.44$, i.e., the risk during this period was 1.44 times greater than the risk for all periods combined. Additionally, we compared any two groups in a specific dimension by dividing their RRs. For example, for the period effect of urban males, the risk of lung cancer mortality in 2013 was 2.09 $\left(\exp\left(\beta_{2013}-\beta_{mean}\right)-\left(\beta_{1988}-\beta_{mean}\right)=\exp\left(\beta_{2013}-\beta_{1988}\right)\approx 2.09\right)$ times the risk in 1988; hence, from 1988 to 2013, the lung cancer mortality risk increased by 1.09-fold.

The four populations had similar life-course patterns, indicating that lung cancer mortality increased with age. However, Figure 2a differs from Figure 1 in that no marked decreasing trend is shown for the elderly in any of the four populations. The bias in Figure 1 is assumed to be due to the mixed effects of one or both of the other two factors (period and cohort), which exhibited relatively strong declining trends in these groups. Therefore, the true age effect on lung cancer mortality was likely to increase monotonically with advancing age, at least for individuals aged 20–84, and this phenomenon is consistent with the theory and practice of lung cancer pathology. From ages 20–24 to 80–84, the lung cancer mortality risks increased by 161.42, 175.47, 87.77, and 98.22 times in urban males, urban females, rural males, and rural females, respectively. These four values were calculated by the exact values of the coefficients and thus may slightly differ from the results shown in Table 1, which were rounded to two decimal places. Revealing the flexuous growths for the four populations, the period effects of lung cancer mortality were divided into two patterns—urban and rural patterns. For urban males and females, the RRs stopped increasing and exhibited declining intervals from 1998 to 2003, thereby forming N-shaped polylines, but there was no obvious decline among rural residents. The RRs increased monotonically in rural males from 1988 to 2008 and in rural females from 1988 to 2013. From 1988 to 2013, the lung cancer mortality risks increased by 1.09, 0.71, 1.83, and 1.56 times for urban males, urban females, rural males, and rural females, respectively.

The cohort effects presented overall downward trends for the four populations and were also divided into two patterns—urban and rural—which suggested continuous improvement across the cohorts who were free from or were survivors of lung cancer. The risks of lung cancer mortality increased slightly with birth year for rural residents born from 1909 to 1928 and subsequently decreased with the exception of the 1984–1988 cohort, while the urban cohorts exhibited monotonic decreasing trends in relation to the birth year. From the 1904–1908 to the 1989–1993 cohort, the lung cancer mortality risks decreased by 95%, 91%, 94%, and 89% in urban males, urban females, rural males, and rural females, respectively. The data from the four populations were reported and processed independently; thus, the period and cohort trends, which were very similar in the same administrative regions (urban/rural), were less likely to be coincidences. Further insights into the variations in the trends are needed, and we present a preliminary discussion in the following section.

## 4. Discussion

After comparing several models and methods of obtaining solutions, we employed APC analysis and the IE to examine temporal changes in the Chinese adult mortality from lung cancer over the past three decades. Based on the results, the questions raised in the introduction of this article can now be discussed. First, the findings showed that important features and changes were related to life-course, time period and cohort effects, which differed to some extent from those reported in previous studies. Second, the trend patterns of the period and cohort effects showed a large gaps between urban and rural residents—the patterns were very similar in the same administrative regions for both males and females when we compared the period and cohort effects (Figure 2b,c). Our preliminary conjecture is that the potential influences of period and cohort could be associated with the differences in societies, regions, economy, and national policies, which are often imbalanced between urban and rural areas.

It is known that period and cohort effects are usually under the influence of a complex set of historical events and environmental factors. For changes that are more prominent in certain time periods, such as world wars, economic crises, epidemics and pandemics of infectious diseases, as well as public health interventions, such a shift may in turn induce similar changes in the lives of all individuals at a given point in time and may be considered period effects. The effects of some determinants that arise earlier in the life course and accumulate in different time periods, such as the improvements in education and science and technology, can be reflected in cohort patterns [19,20]. Therefore, the answer to the last question may lie in the aspects that are supposed to be in focus if the government wants to reduce the lung cancer mortality rates.

Air pollution is an independent factor for lung cancer, and its changes over time are likely to leave imprints on the period trend. More than half of lung cancer cases are related to fine particulates in China and other East Asian countries [21], and this may contribute to the rising period trends of lung cancer mortality in China. Guo et al. [22] have found that lung cancer incidence is statistically associated with fine particulate matter (i.e., with aerodynamic diameters not larger than 2.5 μm, or PM_2.5_) using a spatial age-period-cohort model. Due to the rapid industrial expansion and urbanization that has occurred over the last few decades, an increasing occurrence of haze or smog episodes and continuously rising fine particulate matter concentrations have been reported at the national scale in China, especially in the most developed and highly populated city clusters [23,24], similar to the situation in the UK and United States in the 1970s and 1980s. For rural areas, apart from the burning emission of open crops and indoor solid fuels, a large number of peasants devote most of their lives to urban development and industry. Regardless, these individuals are still registered as rural residents of China. Prolonged exposure to high concentrations of open-air pollutants at construction sites and road traffic [25] as well as to occupational pollutants in factories, significantly increase the risk of respiratory diseases, which may contribute to the regional differences in period trends. In recent years, the government has implemented some legislation and measures to monitor, control and prevent air pollution, such as the new national air quality monitoring network (including the additional indexes of PM_2.5_, CO, and O_3_) projected in 2012. However, air pollution is likely to remain one of the most urgent issues to be addressed to control and prevent lung cancer mortality and to achieve sustainable development.

Medical insurance status is strongly associated with cancer/chronic disease mortality after adjusting for lifestyle factors and is another important factor that may influence period trends. Broader insurance coverage and high coverage rates may improve some of the disparate outcomes of cancer patients and reduce the risk of cancer death [26,27,28]. Similarly, a population-based cancer survival study has shown that rural China has a much lower relative 5-year survival rate than urban China, which could be explained by the limited medical resources, low rates of detection and increased diagnosis of patients with late stage cancers [29,30]. Since the 1990s, China has established three important medical security systems for urban and rural residents. The Decision of the State Council on Setting up Basic Medical Insurance System for Staff Members and Workers in Cities and Towns (No. 44 Document in 1998 of the State Council) was enacted on 14 December 1998, which brought basic medical insurance to urban employees in all sectors. The time of its announcement and implementation corresponded to the only remission period in lung cancer mortality rates amount urban residents, occurring from 1998 to 2003. However, the two later important medical insurance policies on lung cancer mortality did not appear to have pronounced imprints on the period trends of lung cancer mortality. Therefore, the government should provide more support for the medical security system, and further research is needed to confirm the association between medical insurance and lung cancer mortality.

As we know, tobacco smoking is the leading cause of lung cancer. APC analyses in a number of European countries have indicated that cohort patterns are strongly related to smoking rates among adults with lung cancer [19,31,32], and the cohort effect observed for women born since 1940 suggests the start of a lung cancer epidemic associated with a higher prevalence of female smokers in Australia [33]. Thus far, little attention has been devoted to the possible factors involved in the cohort-related mechanisms of lung cancer mortality compared to period effects in China. In this study, we found a significant declining trend due to birth cohort that may be related to the changes in smoking prevalence. Four national-scale tobacco surveys among individuals aged 15 and above in China were conducted in 1984, 1996, 2002, and 2010, and the survey data indicated gradual decreases in the tobacco smoking rates for both males and females over the last two decades (see Appendix Table A1), which may have contributed to the decreased cohort trends. Moreover, improving medical technology, education, and nutritional and exercise levels are also feasible options for controlling lung cancer mortality rates [32,34,35,36,37]. In particular, metformin, a hypoglycemic agent for type 2 diabetes, may be a promising chemoprevention agent for lung cancer prevention. Epidemiologic studies suggest that metformin is associated with lower risk of lung cancer [2,38], colorectal cancer [39], and liver cancer [40] among patients with type 2 diabetes; furthermore, metformin therapy might prolong survival for certain cancer sites [3], though the data from previous epidemiologic studies were highly heterogeneous [3] and validity may be harmed to some extent due to immortal time bias [38,41].

Nonetheless, the battle against lung cancer remains a lengthy one. The effects of these changes are unlikely to manifest until members of cohorts who have spent their early lives in such environments reach an age at which they become vulnerable to chronic diseases [42], and new cohorts are more likely to embrace these changes than older ones, who tend to resist social change [20]. Therefore, although the risk factors of cohorts are decreasing, the epidemic of lung cancer will continue for some time. It is hoped that some Western countries, such as the Netherlands, England and Wales, Finland, and the United States, have reached a point at which their epidemics are finally declining [19,43,44]. China is undergoing arduous and long-term efforts to improve many aspects of lung cancer management, such as environmental protection, medical security, and tobacco control, and so on, and has made great strides. We hope that with the concerted efforts of all sectors, the historically increasing burden of lung cancer can be controlled.

Despite the representativeness of the population and nationwide information, this study has some limitations. First, APC analysis is essentially descriptive, with similar properties as an ecological study, as it considers a community as the observed and analysed unit; this method likely results in ecological fallacies, and we were thus unable to make conclusions regarding the causality of these trends. Nevertheless, we have proposed scientific hypotheses on approaches to reducing lung cancer mortality based on the available data and existing literature, providing a foundation for future research and policies. Second, the available data on influential factors are currently scarce in China. Only four national-scale tobacco sampling surveys have been conducted over the past three decades (Appendix Table A1), and there are no special departments that perform the routine surveillance of tobacco use with a universal coverage of various regions and demographics. Similarly, monitoring of the atmosphere did not include ambient PM_2.5_ related to lung cancer until 2012, and thus, we instead used a model-estimated level of ambient PM_2.5_ (combining remote sensing, global chemical transport models, and ground monitoring air pollution data) reported in recent research. The lack of data makes it somewhat unclear, whether the contribution of this level to lung cancer mortality trends in China can be assessed. Finally several other questions that have not been addressed in this study merit further research. For example, possible explanations for a few of the transitions in cohort trends among rural residents remain uncertain. The identification of potential causes may be helpful to improve prospects for future lung cancer reductions as well. Additionally, case-control studies and cohort studies may improve the evidence of causality between lung cancer mortality and factors such as medical security, smoking, and indoor and outdoor air pollution as well as other possible factors including income, education, and geographic location of residence. 

## 5. Conclusions

To obtain the secular trends in longitudinal survey data, such as trends in the lung cancer mortality of a certain population over several decades, age-period-cohort analysis is one of the best choices to decompose three-dimensional effects and provides an opportunity to rationally relate some environmental effects to the most relevant of the three trends. In this study, we found that the current state of lung cancer mortality remains grim and that the rate is likely to continue increasing for some time in China. Some measures, such as environmental conservation, medical security, and tobacco control, are suggested to be decisive factors for mitigating the risk of lung cancer mortality. There is significant potential for improvement in these aspects, and such improvements should be implemented more vigorously over the long term in China, especially in rural areas.

## Figures and Tables

**Table 1:** Goodness-of-fit in AP (age-period), AC (age-cohort), PC (period-cohort), APC-C (APC models solved with constrained generalized linear model estimator), and APC-IE (age-period-cohort models solved with intrinsic estimator) models for four populations.

*df*	AP	AC	PC	APC-C	APC-IE
	60	48	55	44	44
**Urban Male**					
Deviation	160.89	80.60	478.47	27.40	27.40
AIC	614.30	558.01	941.88	512.80	6.57
BIC	656.72	628.71	996.08	592.93	−164.30
**Urban Female**					
Deviation	108.51	49.36	157.33	24.35	24.35
AIC	510.80	475.65	569.62	458.64	5.88
BIC	553.22	546.35	623.82	538.77	−167.34
**Rural Male**					
Deviation	240.49	108.15	226.33	39.96	39.96
AIC	679.67	571.33	675.50	511.14	6.55
BIC	722.09	642.03	729.71	591.27	−151.74
**Rural Female**					
Deviation	58.07	14.91	31.61	9.11	9.11
AIC	435.50	416.34	419.04	418.53	5.37
BIC	477.92	487.04	473.24	498.66	−182.59

**Table 2:** Lung cancer mortality relative risks due to age, period, and cohort effects.

Factor	Urban Male RR (95% CI)	Urban Female RR (95% CI)	Rural Male RR (95% CI)	Rural Female RR (95% CI)
**Age**				
20–24	0.04 (0.02–0.11)	0.03 (0.01–0.13)	0.06 (0.02–0.17)	0.06 (0.03–0.12)
25–29	0.07 (0.04–0.13)	0.08 (0.04–0.18)	0.08 (0.04–0.18)	0.12 (0.08–0.19)
30–34	0.14 (0.09–0.22)	0.20 (0.12–0.34)	0.20 (0.12–0.32)	0.27 (0.20–0.36)
35–39	0.27 (0.19–0.37)	0.35 (0.24–0.53)	0.29 (0.20–0.44)	0.37 (0.29–0.47)
40–44	0.52 (0.41–0.67)	0.65 (0.47–0.90)	0.58 (0.43–0.78)	0.65 (0.54–0.80)
45–49	0.90 (0.74–1.09)	0.96 (0.74–1.24)	0.94 (0.74–1.20)	0.95 (0.81–1.12)
50–54	1.50 (1.29–1.76)	1.54 (1.25–1.89)	1.51 (1.25–1.83)	1.35 (1.19–1.54)
55–59	2.29 (2.02–2.59)	2.04 (1.73–2.41)	2.13 (1.83–2.48)	1.78 (1.60–1.98)
60–64	3.54 (3.20–3.92)	3.11 (2.72–3.56)	3.01 (2.67–3.39)	2.44 (2.24–2.66)
65–69	5.15 (4.68–5.67)	4.28 (3.78–4.84)	3.93 (3.55–4.36)	3.17 (2.93–3.43)
70–74	6.45 (5.78–7.19)	5.32 (4.64–6.09)	4.98 (4.47–5.56)	4.28 (3.94–4.65)
75–79	6.92 (6.04–7.92)	6.01 (5.10–7.09)	5.53 (4.83–6.32)	4.99 (4.52–5.51)
80–84	6.76 (5.71–8.00)	5.95 (4.85–7.30)	5.62 (4.75–6.65)	5.87 (5.20–6.63)
**Period**				
1988	0.69 (0.62–0.77)	0.78 (0.68–0.89)	0.58 (0.51–0.67)	0.64 (0.58–0.70)
1993	0.87 (0.81–0.93)	0.86 (0.78–0.94)	0.64 (0.59–0.70)	0.68 (0.64–0.73)
1998	1.02 (0.98–1.05)	1.05 (1.00–1.10)	0.85 (0.81–0.90)	0.86 (0.83–0.90)
2003	0.95 (0.91–0.99)	0.92 (0.87–0.98)	1.14 (1.09–1.20)	1.17 (1.12–1.21)
2008	1.20 (1.12–1.29)	1.16 (1.06–1.27)	1.65 (1.53–1.79)	1.39 (1.32–1.47)
2013	1.44 (1.29–1.60)	1.33 (1.16–1.52)	1.65 (1.46–1.86)	1.64 (1.51–1.77)
**Cohort**				
1904–1908	3.00 (2.33–3.85)	2.65 (1.94–3.62)	2.16 (1.61–2.90)	1.79 (1.47–2.18)
1909–1913	3.02 (2.43–3.75)	2.62 (2.01–3.43)	2.06 (1.61–2.64)	1.72 (1.46–2.03)
1914–1918	2.99 (2.46–3.62)	2.56 (2.02–3.24)	2.16 (1.74–2.68)	1.94 (1.69–2.24)
1919–1923	2.74 (2.30–3.27)	2.64 (2.13–3.28)	2.45 (2.03–2.97)	2.09 (1.85–2.37)
1924–1928	2.62 (2.21–3.11)	2.53 (2.06–3.11)	2.75 (2.30–3.30)	2.26 (2.02–2.54)
1929–1933	2.22 (1.87–2.64)	2.26 (1.84–2.79)	2.38 (1.98–2.85)	2.11 (1.88–2.37)
1934–1938	1.66 (1.38–2.00)	1.90 (1.51–2.38)	1.97 (1.62–2.41)	1.95 (1.72–2.22)
1939–1943	1.30 (1.05–1.60)	1.40 (1.09–1.81)	1.57 (1.25–1.96)	1.74 (1.51–2.02)
1944–1948	1.16 (0.91–1.47)	1.09 (0.81–1.46)	1.46 (1.13–1.88)	1.60 (1.35–1.90)
1949–1953	1.15 (0.88–1.51)	1.03 (0.74–1.44)	1.27 (0.95–1.70)	1.46 (1.20–1.77)
1954–1958	1.03 (0.76–1.40)	0.90 (0.62–1.33)	1.01 (0.72–1.42)	1.15 (0.92–1.44)
1959–1963	0.81 (0.57–1.14)	0.66 (0.43–1.02)	0.73 (0.50–1.08)	0.84 (0.65–1.08)
1964–1968	0.57 (0.38–0.86)	0.55 (0.33–0.91)	0.67 (0.43–1.03)	0.73 (0.55–0.97)
1969–1973	0.53 (0.33–0.85)	0.51 (0.28–0.91)	0.49 (0.29–0.83)	0.51 (0.36–0.72)
1974–1978	0.36 (0.19–0.68)	0.34 (0.15–0.74)	0.38 (0.19–0.75)	0.40 (0.26–0.63)
1979–1983	0.27 (0.11–0.68)	0.34 (0.12–0.95)	0.27 (0.10–0.70)	0.30 (0.16–0.54)
1984–1988	0.23 (0.06–0.90)	0.27 (0.05–1.48)	0.33 (0.10–1.15)	0.25 (0.09–0.64)
1989–1993	0.14 (0.01–3.06)	0.24 (0.01–9.94)	0.14 (0.01–3.17)	0.20 (0.03–1.51)

Notes: RR = exp(coefficient), CI = confidence intervals.

## Appendix

**Table A1:** Smoking prevalence (%) (over 15 years of age) in four large-scale tobacco sampling surveys in China.

Sex	Year			
	1984	1996	2002	2010
**Male**	62.0	63.0	57.4	52.9
**Female**	7.0	3.8	2.6	2.4

Notes: For 1984, the results are derived from a 0.5‰ sample tobacco survey based on the third national census in 29 provinces in China. For 1996 and beyond, the results are derived from the Surveillance on Behavioural Risk Factors in China supported by the World Bank’s Loan Project. In total, 145 disease surveillance points were established in 30 provinces in China and three-stage stratified random samples with good representativeness were used.
